# Gut microbiome and metabolome interactions in Crohn’s disease: mechanistic insights into exclusive enteral nutrition-induced remission

**DOI:** 10.3389/fmicb.2025.1616122

**Published:** 2025-07-11

**Authors:** Weiwei Zheng, Quan Zhou, Mengli Xue, Xing Yu, Xue’e Chen, Jintong Chen, Chengdang Wang

**Affiliations:** ^1^Department of Gastroenterology, The First Affiliated Hospital, Fujian Medical University, Fuzhou, China; ^2^Department of Gastroenterology, National Regional Medical Center, Binhai Campus of the First Affiliated Hospital, Fujian Medical University, Fuzhou, China; ^3^Fujian Clinical Research Center for Liver and Intestinal Diseases, Fuzhou, China; ^4^The First Clinical Medical College of Fujian Medical University, Fuzhou, China; ^5^Department of Public Health, Fuzhou Center for Disease Control and Prevention, Fuzhou, China; ^6^The Affiliated Fuzhou Center for Disease Control and Prevention of Fujian Medical University, Fuzhou, China

**Keywords:** Crohn’s disease, exclusive enteral nutrition, gut microbiome, metabolomics, microbiome–metabolome interactions, liquid chromatography–mass spectrometry

## Abstract

**Background:**

Exclusive enteral nutrition (EEN) is a first-line therapeutic approach for inducing remission in Crohn’s disease. However, the underlying mechanisms of its action remain poorly understood. This study aims to investigate the effects of EEN on the gut microbiome and metabolome of CD patients and to elucidate the mechanisms responsible for EEN-induced remission.

**Methods:**

Fecal and blood samples were collected from treatment-naïve CD patients (*n* = 25) both before and after 8 weeks of EEN therapy, as well as from healthy control subjects (*n* = 25). The composition of the gut microbiome was analyzed through 16S rRNA gene sequencing, while metabolomic profiling was conducted using liquid chromatography–mass spectrometry (LC–MS). Functional analysis of microbial pathways was performed utilizing the KEGG Orthology (KO) and MetaCyc databases.

**Results:**

EEN therapy induced significant structural shifts in the gut microbiome, including a reduction in the abundance of pro-inflammatory bacteria such as *Fusobacterium* and *Veillonella*. Metabolomic profiling revealed stage-specific metabolic reprogramming, with notable alterations in phenazine biosynthesis, indole diterpene alkaloid biosynthesis, and sphingolipid metabolism. Functional analyses indicated the activation of energy metabolism pathways and the suppression of pro-inflammatory metabolic pathways. Importantly, EEN therapy was associated with a reduction in oxidative stress and an improvement in gut barrier function.

**Conclusion:**

This study comprehensively integrates microbiome and metabolome analyses, providing new insights into the mechanism of action of EEN therapy in CD. EEN exerts therapeutic effects by restoring metabolic balance and enhancing the integrity of the intestinal barrier, which may be achieved by reducing pro-inflammatory bacteria and activating antioxidant and energy metabolism pathways.

## Introduction

Crohn’s disease (CD), a chronic transmural inflammatory bowel disorder and a principal subtype of inflammatory bowel disease (IBD), has seen a significant rise in incidence globally over the past three decades (van den Heuvel et al., 2017; Mak et al., 2020). Notably, this trend has been especially pronounced in newly industrialized countries in Asia, where the incidence is increasingly “catching up with Western countries” (Ng et al., 2017). This pattern reflects a narrowing of regional disparities in CD prevalence, emphasizing the disease’s growing public health impact worldwide. While current research has identified genetic predispositions (e.g., mutations in the NOD2 and IL23R genes) and aberrant Th1/Th17 immune responses as key pathogenic mechanisms of CD, emerging evidence underscores the critical role of environmental factors and gut microbiome dysbiosis in driving disease initiation and progression (Jostins et al., 2012; Ananthakrishnan et al., 2024). Research indicates that patients with CD exhibit gut microbiota dysbiosis, characterized by a significant reduction in microbial diversity (Sokol et al., 2017; Lewis et al., 2024). Notably, the abundance of pathogenic bacteria, such as *adherent-invasive Escherichia coli*, has increased, while beneficial commensals like *Faecalibacterium prausnitzii* have been depleted (Bustamante and Vidal, 2020; Touch et al., 2022; El Atty et al., 2024). This dysbiotic profile contributes to the development of intestinal inflammation through several interconnected mechanisms: it disrupts epithelial barrier function, activates NF-κB signaling pathways, and triggers excessive production of pro-inflammatory cytokines, including TNF-α and IL-6 (Sokol et al., 2008; Gevers et al., 2014). Additionally, studies have indicated that CD is associated with gut microbial metabolite imbalances, including reduced short-chain fatty acids (SCFAs) and increased levels of secondary bile acids and hydrogen sulfide (Ambrozkiewicz et al., 2020; Kiasat et al., 2024; Luo et al., 2024). These metabolic alterations collectively impair intestinal epithelial energy metabolism, compromise immune regulation, and disrupt oxidative stress homeostasis, thereby establishing a self-perpetuating cycle of microbiome-metabolite-immune dysfunction that sustains chronic intestinal inflammation in CD (Lavelle and Sokol, 2020).

Recent advancements in metabolomics have provided new insights into the pathogenesis of CD. Metabolomic analyses, utilizing mass spectrometry and nuclear magnetic resonance, have revealed significant abnormalities across various metabolic pathways in both fecal and serum samples from CD patients (Liu et al., 2022; Shi et al., 2022). These include disruptions in tryptophan metabolism (characterized by reduced levels of indole derivatives, leading to the suppression of AhR signaling) (Roager and Licht, 2018), bile acid metabolism (with the accumulation of primary bile acids contributing to cytotoxicity) (Franzosa et al., 2019), and impaired fatty acid β-oxidation (Wikoff et al., 2009). In summary, we conclude that the synergistic dysregulation of both the microbiome and metabolome may represent a potential underlying cause for the refractory nature and recurrence of CD (Lloyd-Price et al., 2019).

EEN is widely regarded as the first-line treatment for inducing remission in CD, particularly in pediatric patients (Gurram and Patel, 2019). Studies have shown that the efficacy of EEN is comparable to that of corticosteroids while avoiding the potential side effects associated with corticosteroid use (Ashton et al., 2019). EEN effectively controls inflammation and promotes mucosal healing by reducing the expression of key inflammatory factors, such as IL-6 and TNF-α (Gerasimidis et al., 2014). Additionally, research by Konstantinos Gerasimidis et al. (2014) has demonstrated that EEN treatment typically leads to a reduction in gut microbial diversity, along with changes in the abundance of specific bacterial taxa. Notably, patients who respond to EEN exhibit a decrease in the abundance of protective bacteria, including *Faecalibacterium prausnitzii* and *Bifidobacterium* spp., which may be associated with disease remission.

In summary, the gut microbiota serves as a pivotal interconnector bridging the immune system and enteral nutrition. Furthermore, an in-depth investigation into its complex regulatory mechanisms may provide deeper insights into the interactions and underlying mechanisms among these three systems. This study aims to systematically evaluate the gut microbiome composition and metabolomic profiles of CD patients before and after EEN therapy, as well as in healthy controls, through 16S rRNA gene sequencing and untargeted metabolomics. The primary objectives are to clarify how EEN modulates intestinal microbial ecosystems and metabolic networks in CD patients, and to explore the potential mechanisms underlying microbiome-metabolite interactions during EEN-induced disease remission. These findings will provide a scientific foundation for refining nutritional therapeutic strategies in CD management.

## Materials and methods

### Patients

Sample collection and nutritional intervention studies were conducted at the First Affiliated Hospital of Fujian Medical University, involving patients diagnosed with CD. The recruitment process adhered to predefined inclusion and exclusion criteria. Inclusion criteria: The case group consisted of patients with active CD who were presenting for the first time and met the World Health Organization (WHO) diagnostic criteria (Guarner et al., 2024): CDAI >150 and <400; age ≥14 years. Exclusion criteria: (i) CD with intestinal obstruction, as it alters gut transit time and microbiota; (ii) CD with high—flow fistula, which affects gut continuity and function; (iii) patients treated with glucocorticoids, immunosuppressants, biologics, antimicrobials, or probiotics within 3 months before enrolment, as these can affect the gut microbiota. Healthy controls were age—sex matched individuals without digestive diseases or severe organ insufficiency. The healthy control group was selected from age- and sex-matched individuals without digestive system diseases or severe organ insufficiency. All procedures followed the ethical guidelines approved by the Ethics Committee of Fujian Medical University [Approval number: MRCTA, ECFAH of FMU (2022) 317]. All patients provided written informed consent.

### Experimental method

The CD group was administered EEN therapy (Peptisorb Liquid, Nutricia) via nasal feeding, according to the EEN requirement of 30–35 kcal/day during the active phase of the disease. Patients in the CD group received all their caloric and nutritional needs solely through EEN and were prohibited from consuming any other sustenance during the feeding period. The duration of the EEN course was 8 weeks in total. Clinical data, along with stool and blood specimens, were collected from the healthy control group, the CD group before EEN treatment, and the CD group after EEN treatment. The collected clinical indicators included BMI, biochemical and hematological parameters. All samples were immediately stored in a standard −80°C freezer for long-term preservation until further analysis.

### 16S rRNA

Fecal samples were stored at 4°C and transferred to −80°C within 15 min. DNA was extracted using the QIAamp DNA Stool Mini Kit (QIAGEN, Germany). PCR amplified the 16S rRNA gene V3-V4 region with primers: forward 5′-CCTACGGGNGGCWGCAG-3′ and reverse 5′-GACTACHVGGGTATCTAATCC-3′. The 25 μL PCR mix contained 12.5 μL 2x KAPA HiFi HotStart ReadyMix, 0.5 μL primers (10 μM), 10 μL DNA, and 1.5 μL ddH2O. Products were confirmed by agarose gel electrophoresis and purified with AMPure XP Beads.

The raw microbial sequencing data were imported into QIIME2-2022.08. Specifically, the QIIME2 import tool was utilized to process the 16S rRNA data, resulting in the generation of the demux.qza file. Subsequent parameter optimization was performed based on the demux.qzv file, which was visualized from the demux.qza file. The DADA2 algorithm was then employed for denoising to eliminate low-quality reads from the sequencing data. We filtered out ASV reads that constituted less than 5% of the sample reads. For the retained high-quality ASVs, the Naïve Bayes classifier^1^ was applied to annotate and classify the filtered amplicon sequence variants (ASVs). These annotated ASVs were subsequently used to construct phylogenetic trees.

### Peripheral blood metabolomics analysis

Metabolites were extracted from frozen blood samples using LC–MS. The extraction protocol was as follows: 200 μL of blood was combined with 800 μL of pre-chilled methanol:acetonitrile (1:1) and vortexed for 1 min. The mixture was then incubated at −20°C for 1 h, followed by centrifugation at 12,000×*g* for 15 min. The supernatant was carefully collected, dried under a vacuum concentrator, and reconstituted in 100 μL of a 50% methanol solution for LC–MS analysis. For the LC–MS analysis, an Agilent 1290 Infinity II liquid chromatograph coupled to an Agilent 6545 Q-TOF mass spectrometer was used. Chromatographic separation was performed on an Agilent Poroshell 120 EC-C18 column (2.1 × 100 mm, 2.7 μm) maintained at 40°C. The mobile phase consisted of 0.1% formic acid in water (A) and 0.1% formic acid in acetonitrile (B). The gradient elution program was set as follows: 0–2 min, 95% A; 2–8 min, 95–5% A; 8–10 min, 5% A; 10–12 min, 5–95% A; 12–15 min, 95% A. The flow rate was maintained at 0.4 mL/min. The mass spectrometer was operated in positive and negative ion modes with the following parameters: gas temperature, 300°C; gas flow, 11 L/min; nebulizer pressure, 45 psi; sheath gas temperature, 350°C; sheath gas flow, 12 L/min; capillary voltage, 3,500 V; fragmentor voltage, 130 V; skimmer voltage, 60 V; octopole RF voltage, 750 V. The mass range was set from m/z 100 to 1700. Data acquisition and analysis were performed using Agilent MassHunter Workstation Software. Four LC–MS methods were employed to measure the gut metabolome in fecal samples, covering polar metabolites, lipids, free fatty acids, and bile acids. MS data acquisition involved metabolite library construction with the NEBNext Ultra Metabolite Library Prep Kit (NEB, USA), following the kit’s protocol for extraction, enrichment, and purification. The quality of the library was assessed using the Agilent 2100 Bioanalyzer prior to sequencing on an LC–MS platform for high-resolution metabolite profiles.

### Functional analysis

We employed the Phylogenetic Investigation of Unobserved States (PICRUSt2) algorithm to predict metabolic pathways using the MetaCyc metabolic pathway database, a comprehensive repository of experimentally validated metabolic pathways across all domains of life.^2^ To examine differences in microbial functional profiles among the healthy control, post-treatment, and pre-treatment groups, we applied the DESeq2 differential expression analysis algorithm. A false discovery rate (FDR) threshold of <0.05 and a log2-fold change (log2FC) threshold were set to identify microbial functions that exhibited statistically significant differences between the groups. This approach enabled a systematic evaluation of the functional shifts in microbial communities associated with treatment effects.

### Statistical analysis

Data processing and analysis were performed using GraphPad Prism software. Results are presented as the mean ± standard error of the mean unless otherwise specified. Comparisons between CD patients before and after EEN therapy were conducted using either a *t*-test or one-way ANOVA, with *p* values <0.05 considered statistically significant. The results were then integrated with gut microbiome characteristics and metabolite data to explore potential mechanistic associations.

## Results

### Exclusive enteral nutrition therapy in CD patients leads to significant improvements in nutritional status

A total of 18 CD patients after EEN treatment, 25 CD patients before EEN treatment, and 24 healthy controls were included in this study. EEN therapy led to significant improvements in various biochemical and hematological parameters in CD patients (Table 1). Notably, albumin levels increased significantly (*p* < 0.001), indicating enhanced nutritional status. Inflammatory markers, including erythrocyte sedimentation rate (ESR) and C-reactive protein (CRP), showed significant reductions (both *p* < 0.001), reflecting a decrease in systemic inflammation. Furthermore, the Crohn’s Disease Activity Index (CDAI) score, which assesses disease activity, decreased significantly (*p* < 0.001), suggesting clinical improvement. These findings underscore the efficacy of EEN in managing CD by improving both nutritional and inflammatory profiles.

**Table 1:** Clinical and laboratory parameters of CD patients before and after enteral nutrition therapy.

Parameter	Before treatment	After treatment	*Z* score	*p* Value
Albumin (g/L)	35.85 (31.13 ~ 40.75)	41.65 (36.63 ~ 44.98)	−5.075	<0.001
BMI	17.1 (16 ~ 18.33)	17.435 (15.73 ~ 18.73)	−1.794	0.073
ESR (mm/h)	62.5 (46.25 ~ 84.75)	23 (15.25 ~ 45.25)	−4.463	<0.001
CRP (mg/L)	45.82 (23.25 ~ 69.69)	1.76 (0.70 ~ 14.38)	−5.232	<0.001
FC			−4.803	<0.001
Negative	0	22		
Weak positive	6	9		
Positive	30	5		
CDAI score	210.4 (151.5 ~ 272.7)	101.5 (45.5 ~ 156.8)	−5.159	<0.001
White blood cells	7.94 (6.36 ~ 9.77)	5.87 (4.94 ~ 7.09)	−3.724	<0.001
Red blood cells	4.46 (3.98 ~ 4.86)	4.45 (4.21 ~ 4.95)	−1.312	0.19
Hemoglobin (g/L)	106 (93 ~ 121)	116.5 (106.3 ~ 131.5)	−4.072	<0.001
Platelets	438.5 (371.3 ~ 515.3)	317.5 (227.0 ~ 369.3)	−4.98	<0.001

This table summarizes the median values and interquartile ranges (IQR) of various clinical and laboratory parameters measured in CD patients before and after EEN therapy. The parameters include Albumin, Body Mass Index (BMI), Erythrocyte Sedimentation Rate (ESR), C-Reactive Protein (CRP), Fecal Calprotectin (FC), Crohn’s Disease Activity Index (CDAI) score, Leukocytes, Erythrocytes, Hemoglobin, and Platelets. The *Z*-values and *p*-values indicate the statistical significance of the changes observed post-therapy compared to pre-therapy measurements. Significant improvements were noted in Albumin, ESR, CRP, CDAI score, Leukocytes, Hemoglobin, and Platelets, suggesting a positive response to the EEN therapy.

### Exclusive enteral nutrition therapy induces structural shifts in the gut microbiome of CD patients

**Figure 1:**
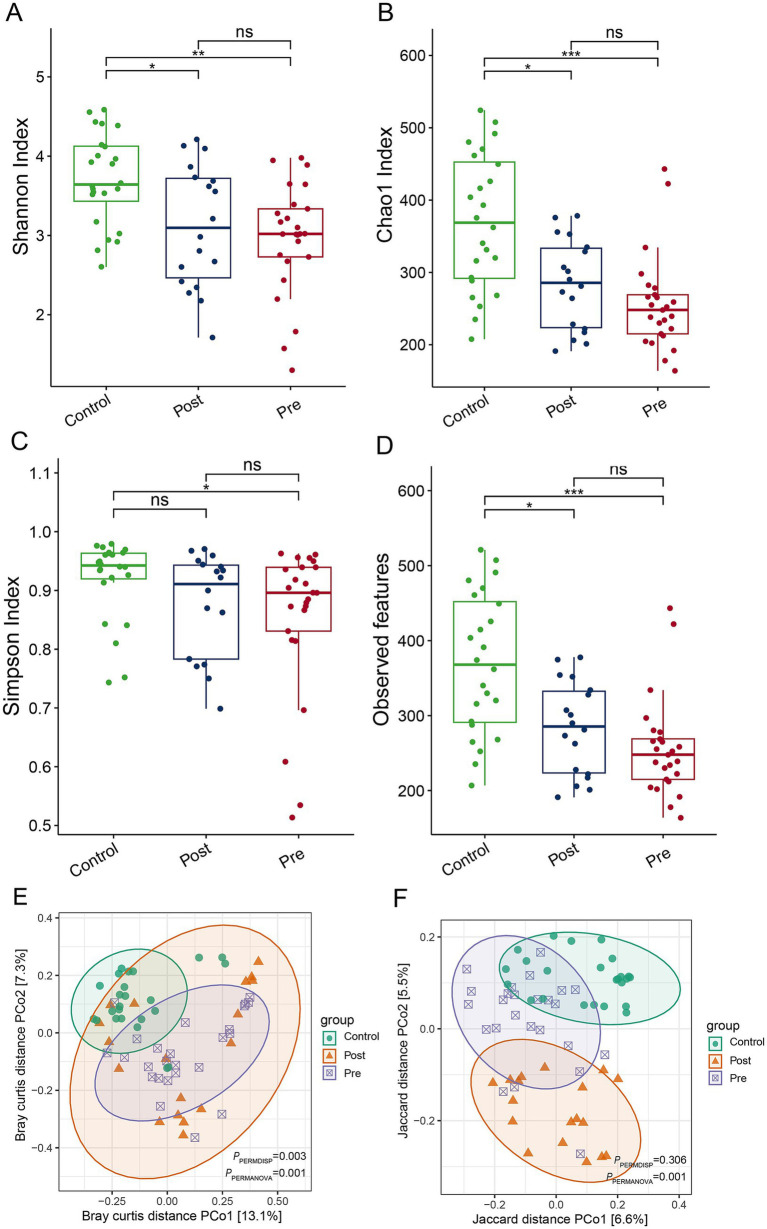
Impact of enteral nutrition therapy on gut microbiome diversity in CD patients. **(A)** Shannon index; **(B)** Chao 1 index; **(C)** Simpson index **(D)** observed ASVs; **(E)** PCoA based on Bray–Curtis distances; **(F)** PCoA based on Jaccard distances.

EEN therapy induces significant alterations in the gut microbiome structure of CD patients, despite no notable change in overall alpha diversity. Indices such as the Shannon index, Chao 1 index, and observed features revealed that the post-treatment and pre-treatment groups exhibited significantly reduced alpha diversity compared to the healthy control group (Figures 1A–C). Additionally, the Simpson index indicated that the pre-treatment group had substantially lower alpha diversity than the healthy control group (Figure 1D). Principal coordinate analysis (PCoA), based on Bray-Curtis and Jaccard distances, demonstrated significant differences in beta diversity among the healthy control, post-treatment, and pre-treatment groups (Figures 1E,F). These findings underscore the impact of EEN on the gut microbiome structure in CD patients, emphasizing its therapeutic potential in modulating the gut microbiome.

### Microbial composition characteristics of different treatment stages

**Figure 2:**
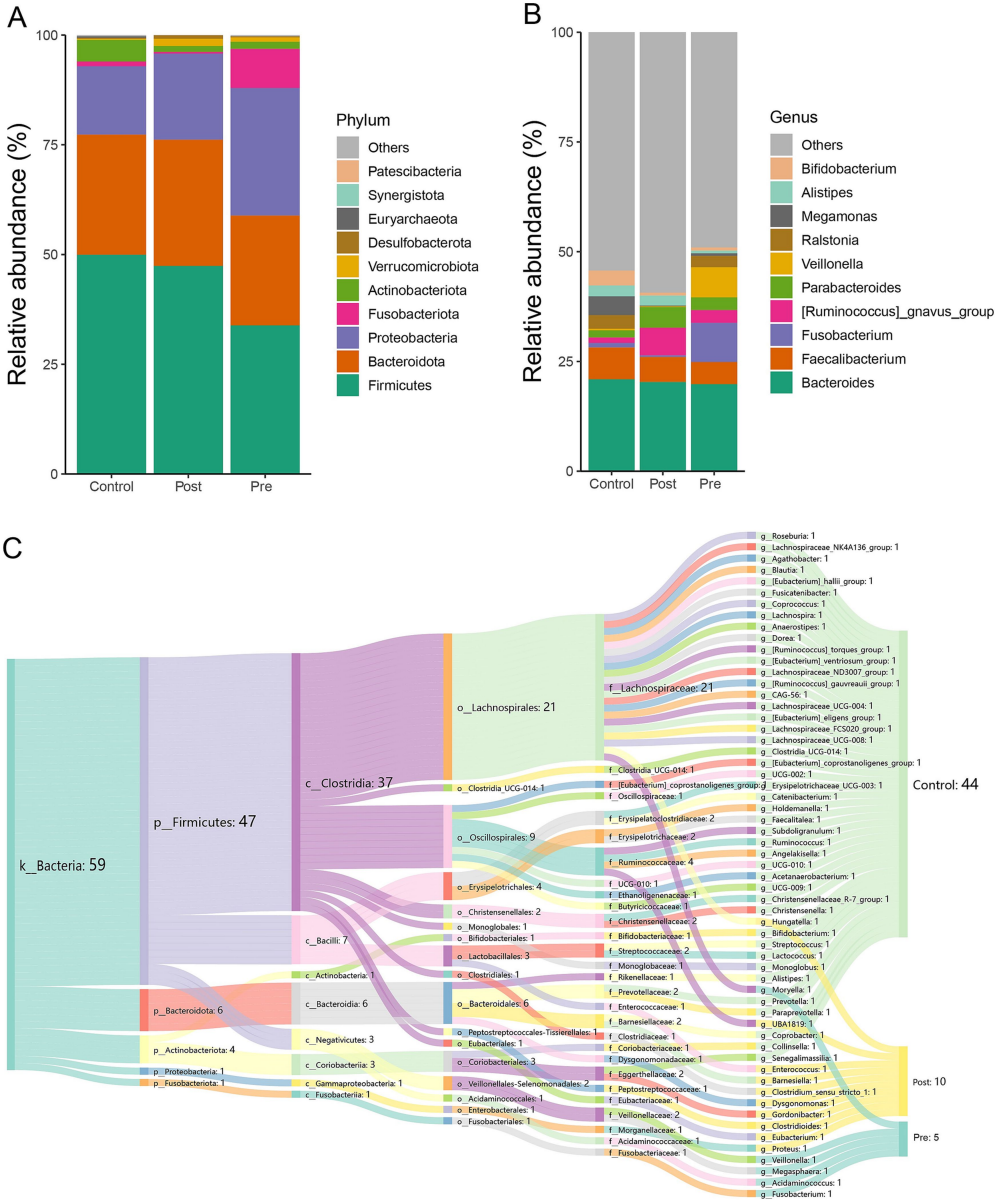
Microbial composition characteristics of different treatment stages. **(A)** The relative abundance stacked barplot of top 10 phyla; **(B)** the relative abundance stacked barplot of top 10 families; **(C)** gut differential microbes among the three groups.

At the phylum level, the dominant microbial phyla across the healthy control, post-treatment, and pre-treatment groups included *Firmicutes*, *Bacteroidota*, *Proteobacteria*, *Fusobacteriota*, *Actinobacteriota*, *Verrucomicrobiota*, *Desulfobacterota*, *Euryarchaeota*, *Synergistota*, and *Patescibacteria* (Figure 2A). Analysis of microbial community abundance revealed that the top 10 genera across the three groups were *Bacteroides*, *Faecalibacterium*, *Fusobacterium*, *Ruminococcus gnavus*, *Parabacteroides*, *Veillonella, Ralstonia*, *Megamonas*, *Alistipes*, *Hungatella*, *Eubacterium*, *Gordonibacter*, *Dysgonomonas*, and *Bifidobacterium* (Figure 2B).

LEfSe analysis at the genus level revealed significant differences in microbial composition among the three groups. The healthy control group showed enrichment of 44 microbial taxa, with beneficial genera such as *Bifidobacterium*, *Roseburia*, and *Levilactobacillus cerevisiae* standing out. These beneficial genera are known to enhance immunity and produce short-chain fatty acids, promoting gut health. The post-treatment group exhibited enrichment of 10 genera, which may also have beneficial effects, such as *Eubacterium*, *Gordonibacter*, and *Barnesiella*. These bacteria can potentially contribute to anti-inflammatory effects and gut homeostasis. The pre-treatment group had higher abundances of *Fusobacterium*, *Veillonella*, *Acidaminococcus*, *Megasphaera*, and *Moryella*, which are generally considered harmful or pathogenic and are often associated with gastrointestinal disorders and infections (Figure 2C). These findings highlight distinct microbial signatures associated with treatment status and health controls.

### Analysis of functional differences of gut microbiota at different stages of treatment

**Figure 3:**
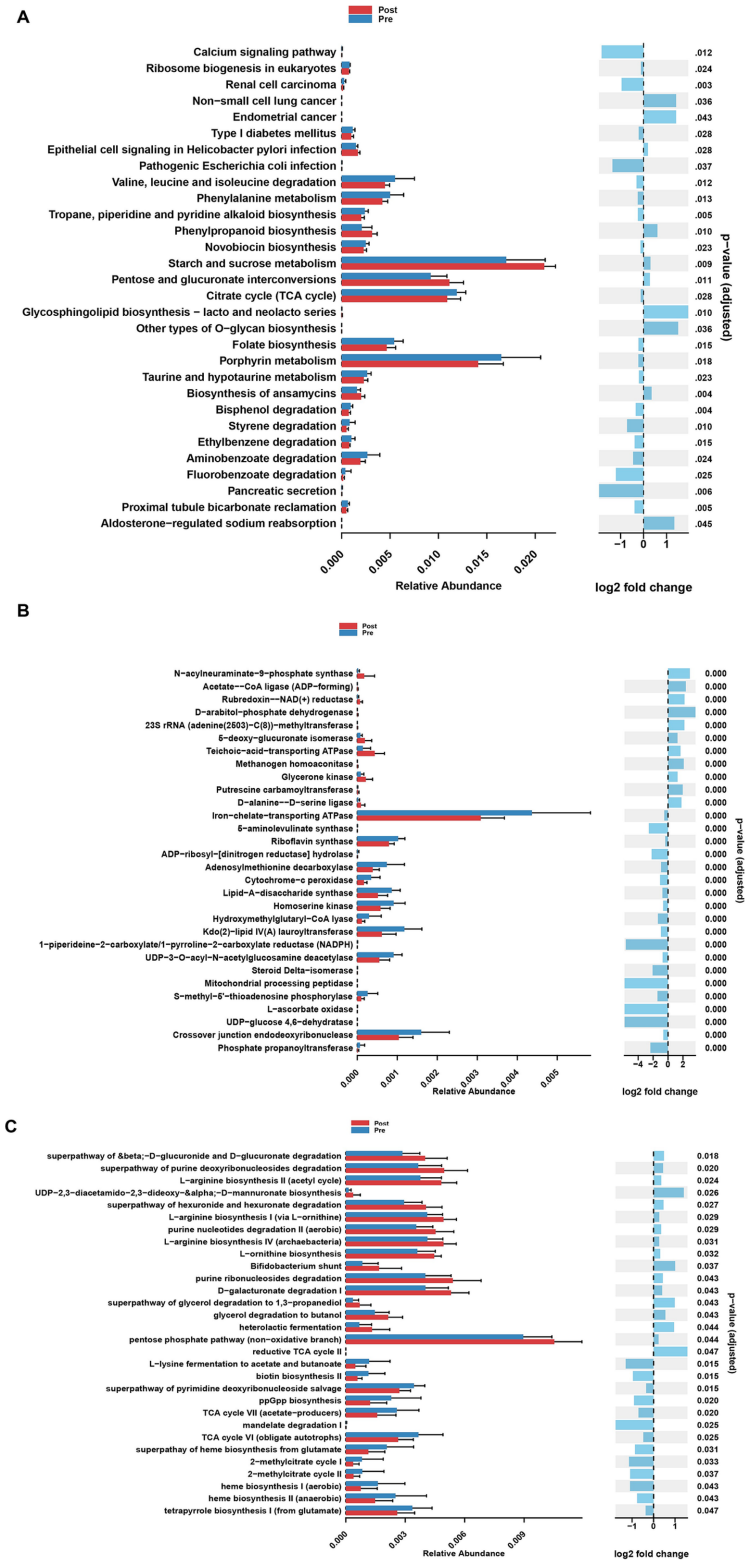
Analysis of functional differences of gut microbiota at different stages of treatment. **(A)** Differential KEGG Orthology (KO) functions of gut microbes between pre-treatment group and post-treatment group. **(B)** Differential enzyme functions of gut microbes between pre-treatment group and post-treatment group. **(C)** Differential MetaCyc pathways functions of gut microbes between pre-treatment group and post-treatment group.

At the KO pathway level, the post-treatment group exhibited significant activation of energy metabolism pathways, including ABC transporters, the pentose phosphate pathway, and glycolysis. In contrast, the pre-treatment group was characterized by predominant alterations in butanoate metabolism and glutathione metabolism (Figure 3A). EC enzyme analysis revealed a marked upregulation of 13 enzymes, including L-seryl-tRNA(Sec) selenium transferase, in the post-treatment group, alongside specific modifications in 19 enzymes, such as iron-chelate-transporting ATPase, following treatment (Figure 3B). MetaCyc pathway analysis further demonstrated substantial enhancement of nucleotide biosynthesis pathways, particularly the biosynthesis of adenosine and guanosine deoxyribonucleotides, in the post-treatment group, concomitant with the suppression of biotin and folate biosynthesis pathways (Figure 3C).

### Metabolomic profiling distinguishes treatment effects across patient group

**Figure 4:**
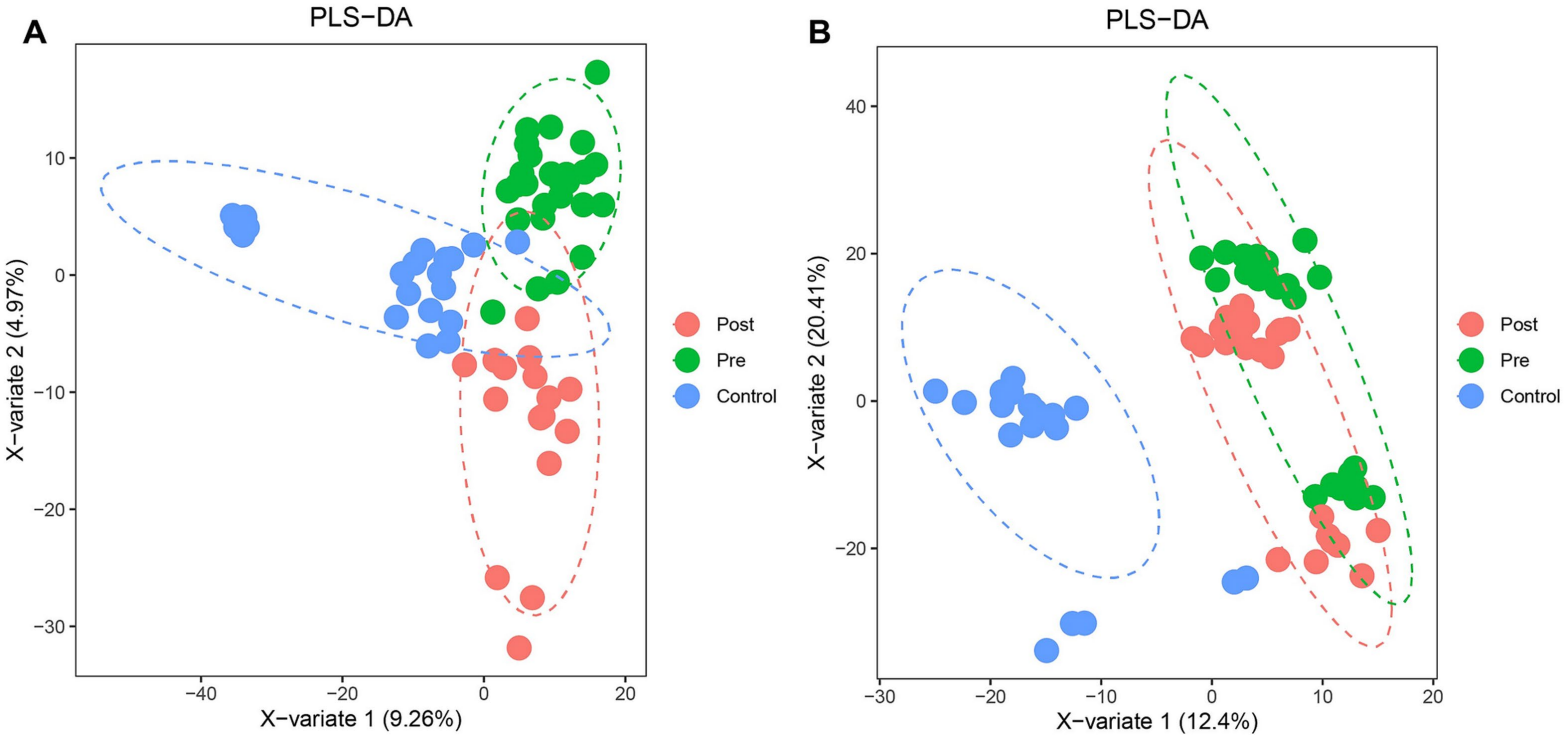
PLS-DA analysis of microbial community structure changes in Crohn’s patients before and after ENN treatment. **(A)** PLS-DA model showing the classification of fecal samples from healthy controls, pre-treatment, and post-treatment CD patients; **(B)** PLS-DA model showing the classification of plasma samples from healthy controls, pre-treatment, and post-treatment CD patients.

**Figure 5:**
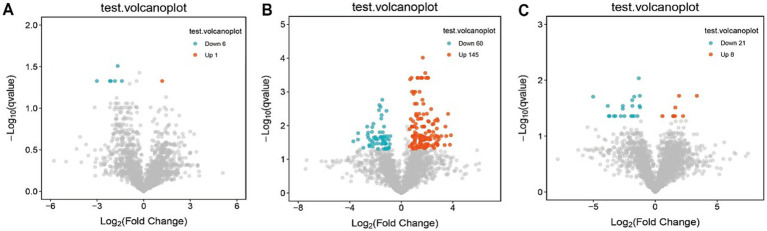
Volcano plot of differential metabolites in fecal. **(A)** Volcano plots show differential metabolites in the pre-treatment and post-treatment groups; **(B)** volcano plots show differential metabolites in the pre-treatment and healthy control groups; **(C)** volcano plots show differential metabolites in the post-treatment and healthy control groups.

**Figure 6:**
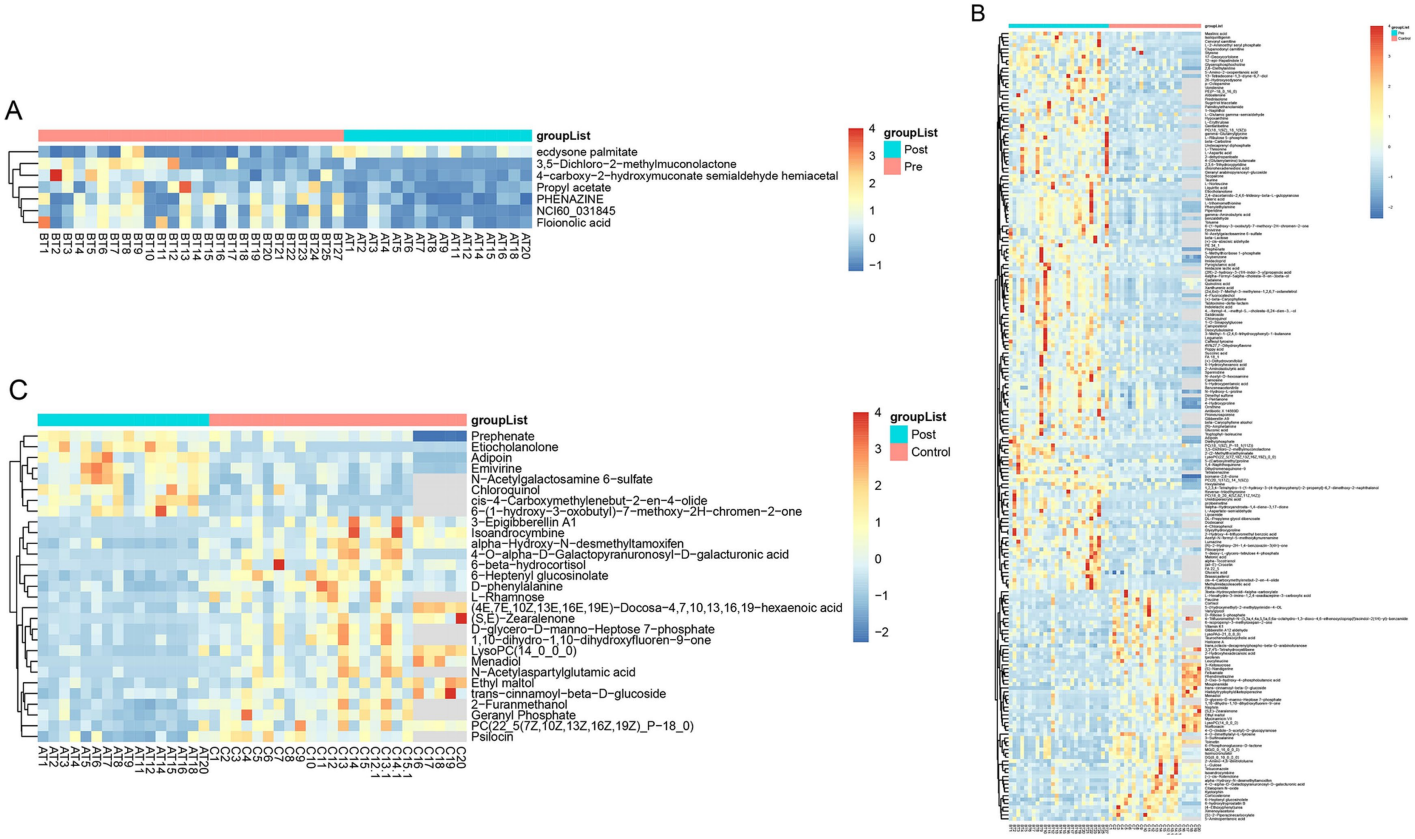
Heatmap of differential metabolites in fecal. **(A)** Heatmap show differential metabolites in the pre-treatment and post-treatment groups; **(B)** heatmap show differential metabolites in the pre-treatment and healthy control groups; **(C)** heatmap show differential metabolites in the post-treatment and healthy control groups.

Partial least squares-discriminant analysis (PLS-DA) was conducted to analyze the fecal metabolomic profiles of the healthy control, post-treatment, and pre-treatment groups. The PLS-DA score plot revealed that post-treatment samples were positioned between the pre-treatment and healthy control groups, indicating a partial shift in the metabolic profile toward a healthier state following therapy (Figure 4A). Volcano plot analysis further demonstrated significant alterations in fecal metabolites. Compared to the pre-treatment group, the post-treatment group exhibited downregulation of six metabolites (3,5-Dichloro-2-methylmuconolactone, NCI60_031845, Floionolic acid, PHENAZINE, 4-carboxy-2-hydroxymuconate semialdehyde hemiacetal, and Propyl acetate) and upregulation of one metabolite (ecdysone palmitate) (Figures 5A, 6A). When compared to the healthy control group, the pre-treatment group showed a downregulation of 60 metabolites and an upregulation of 145 metabolites (Figures 5B, 6B). In contrast, the post-treatment group demonstrated a downregulation of 21 metabolites and an upregulation of eight metabolites relative to healthy controls (Figures 5C, 6C). These findings highlight the metabolic shifts associated with EEN therapy and suggest its potential role in modulating the gut metabolome toward a healthier profile.

**Figure 7:**
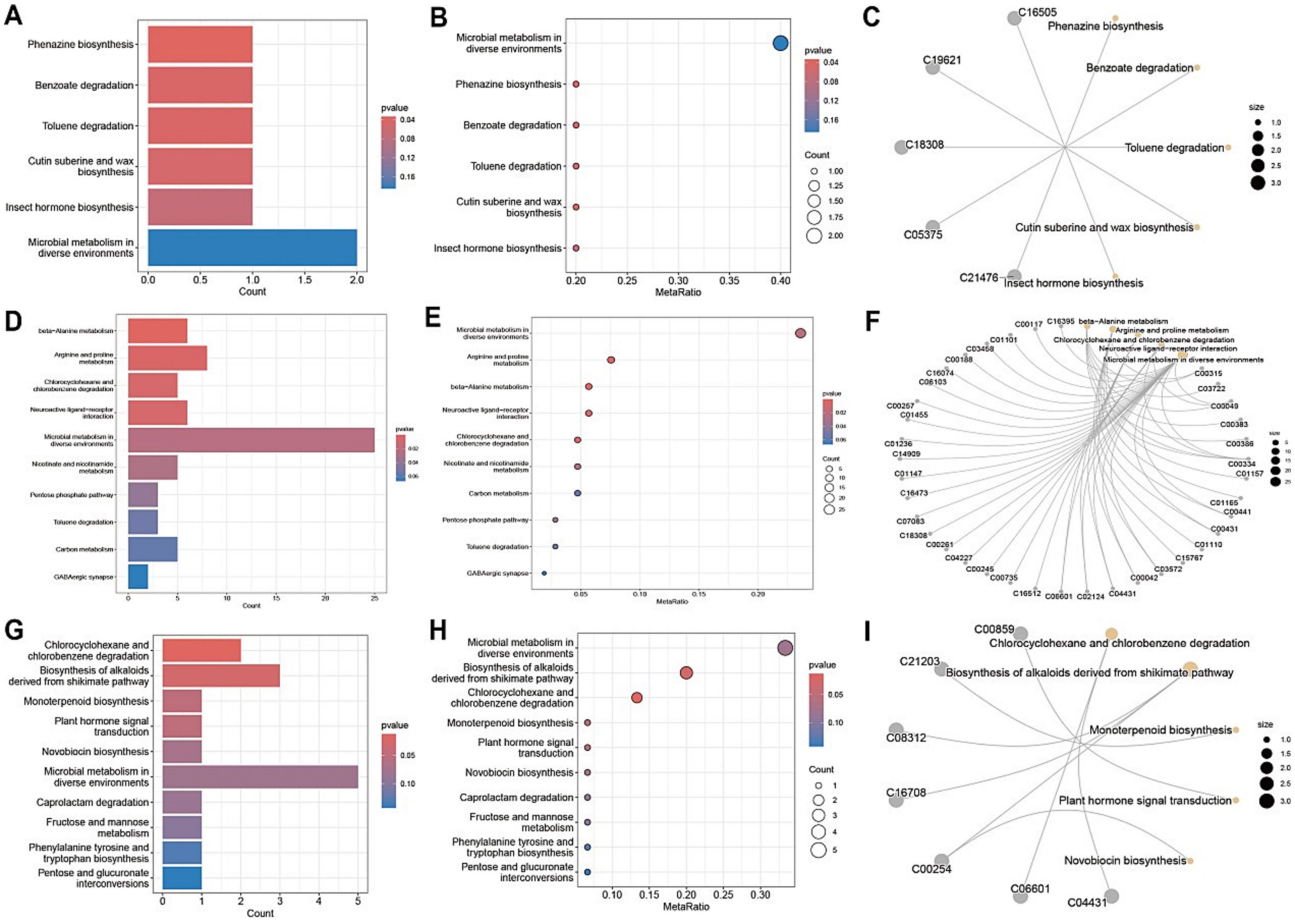
Metabolic pathway analysis of fecal samples. **(A,D,G)** Metabolic pathway impact factor histogram. The vertical coordinates represent the metabolic pathways and the horizontal coordinates represent the enrichment of different metabolic pathways Impact values. **(B,E,H)** Metabolic pathway impact factor bubble diagram. **(C,F,I)** Metabolite and metabolic pathway network diagram. **(A–C)** shows the comparison between the post-treatment group and the pre-treatment group. **(D–F)** shows the comparison between the pre-treatment group and the healthy control group. **(G–I)** shows the post-treatment group compared to the healthy control group.

A comprehensive metabolic pathway analysis of fecal samples from CD patients undergoing EEN therapy revealed distinct metabolic patterns across different phases of treatment. In the comparison between the post-treatment and pre-treatment groups (Figures 7A–C), significant alterations were observed in key microbial metabolic pathways, including phenazine biosynthesis, benzoate degradation, and toluene degradation, along with notable fold changes in cutin, suberin, and wax biosynthesis. The comparison between the pre-treatment group and healthy controls (Figures 7D–F) demonstrated profound baseline disturbances in microbial metabolism, particularly affecting secondary metabolite biosynthesis and specific pathways such as caprolactam degradation. The post-treatment versus healthy control comparison (Figures 7G–I) revealed persistent yet attenuated metabolic dysregulation, with incomplete normalization of phenazine biosynthesis and toluene degradation pathways, as well as residual abnormalities in cutin/suberine biosynthesis. Network analyses (Figures 7C,F,I) illustrated the complex interactions between these metabolic pathways, highlighting the extensive and specific nature of EEN-induced metabolic remodeling.

**Figure 8:**
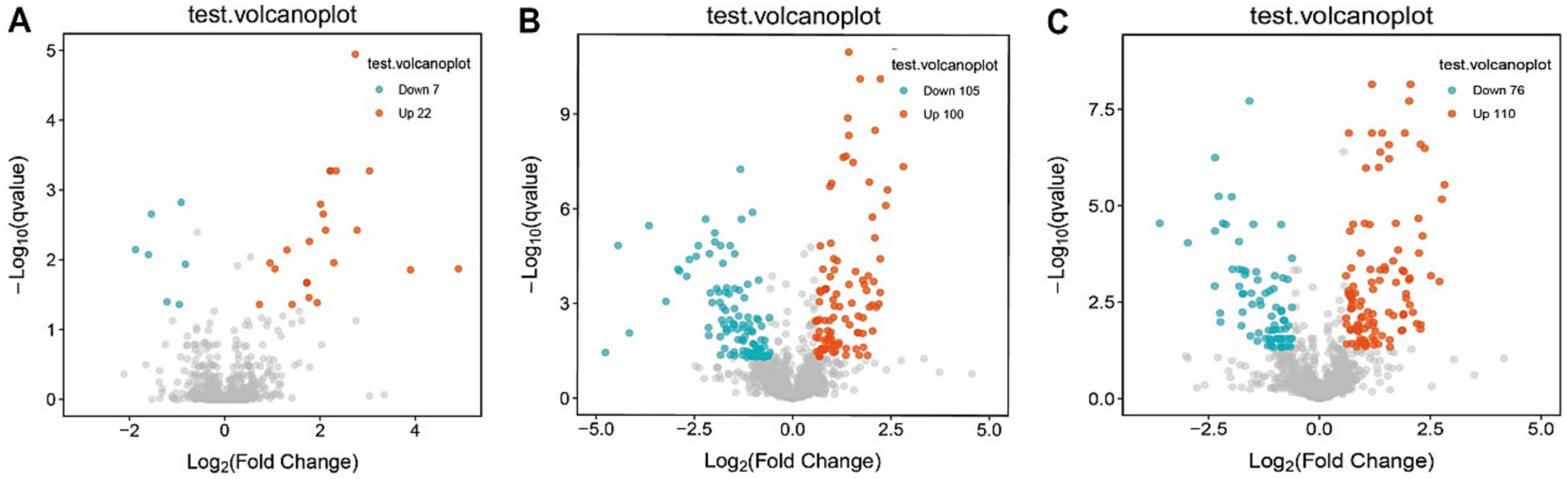
Volcano plot of differential metabolites in plasma. **(A)** Volcano plots show differential metabolites in the pre-treatment and post-treatment groups; **(B)** volcano plots show differential metabolites in the pre-treatment and healthy control groups; **(C)** volcano plots show differential metabolites in the post-treatment and healthy control groups.

**Figure 9:**
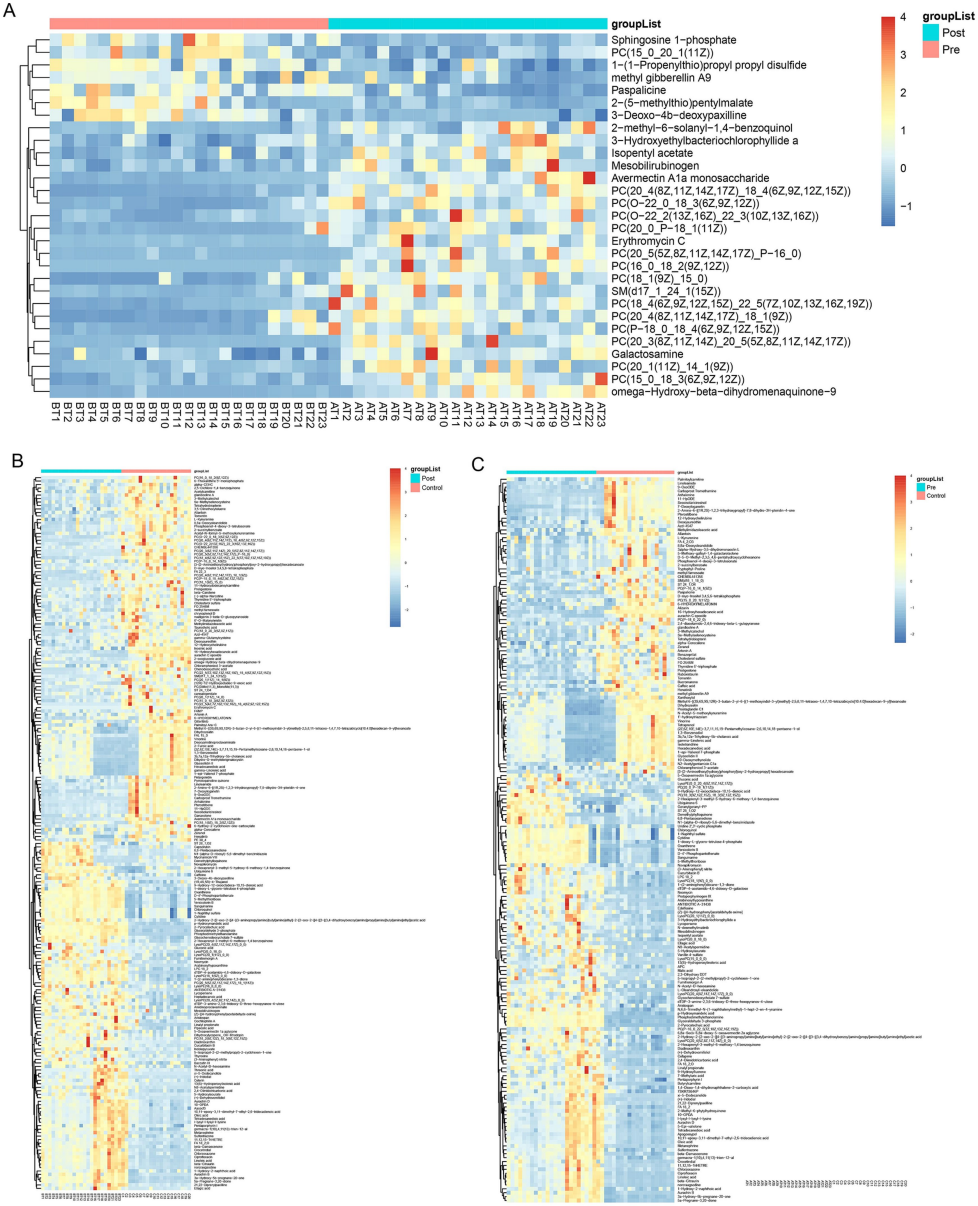
Heatmap of differential metabolites in plasma. **(A)** Heatmap show differential metabolites in the pre-treatment and post-treatment groups; **(B)** heatmap show differential metabolites in the pre-treatment and healthy control groups; **(C)** heatmap show differential metabolites in the post-treatment and healthy control groups.

Consistent patterns were observed in blood samples from the same cohort using PLS-DA analysis (Figure 4B). Volcano plot analysis of fecal metabolites revealed significant alterations: compared to the pre-treatment group, the post-treatment group exhibited 7 downregulated metabolites [Sphingosine 1-phosphate, 2-(5-methylthio) pentylmalate, 1-(1-Propenylthio) propyl propyl disulfide, PC (15:0/20:1(11Z)), Paspalicine, 3-Deoxo-4b-deoxypaxilline, and methyl gibberellin A9] and 22 upregulated metabolites [PC (16:0/18:2(9Z,12Z)), Erythromycin C, omega-Hydroxy-beta-dihydromenaquinone-9, etc.] (Figures 8A, 9A). More substantial differences were observed when comparing the pre-treatment group to healthy controls, with 105 downregulated and 100 upregulated metabolites (Figures 8B, 9B). In contrast, the post-treatment group showed 76 downregulated and 110 upregulated metabolites (Figures 8C, 9C).

**Figure 10:**
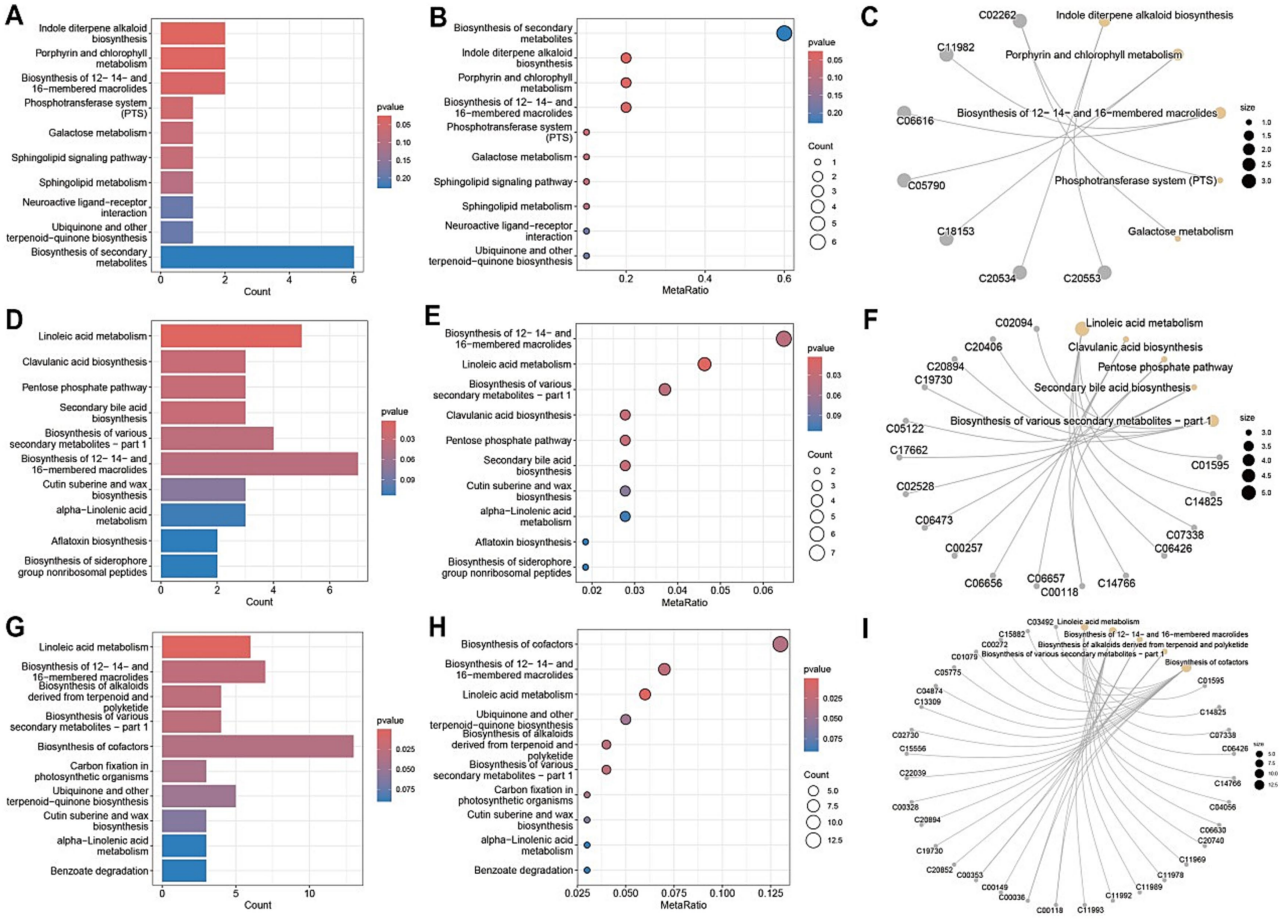
Metabolic pathway analysis of plasma samples. **(A,D,G)** Metabolic pathway impact factor histogram. The vertical coordinates represent the metabolic pathways and the horizontal coordinates represent the enrichment of different metabolic pathways impact values. **(B,E,H)** Metabolic pathway impact factor bubble diagram. **(C,F,I)** Metabolite and metabolic pathway network diagram. **(A–C)** shows the comparison between the post-treatment group and the pre-treatment group. **(D–F)** shows the comparison between the pre-treatment group and the healthy control group. **(G–I)** shows the post-treatment group compared to the healthy control group.

Metabolic pathway analysis of blood samples from CD patients undergoing EEN therapy revealed three distinct patterns of metabolic remodeling across different treatment phases. In the post-treatment versus pre-treatment comparison (Figures 10A–C), significant upregulation was observed in specialized metabolic pathways, including indole diterpene alkaloid biosynthesis and porphyrin/chlorophyll metabolism, alongside the activation of the phosphotransferase system (PTS) and sphingolipid signaling pathways. The pre-treatment versus healthy control comparison (Figures 10D–F) demonstrated marked dysregulation in key metabolic processes, particularly linoleic acid metabolism, and the pentose phosphate pathway, accompanied by enhanced biosynthesis of various secondary metabolites. Network analyses (Figures 10C,I) revealed intricate metabolic interactions, underscoring the central role of sphingolipid metabolism in the treatment response and its strong connectivity with neuroactive ligand-receptor interactions and secondary metabolite biosynthesis pathways. The post-treatment versus control comparison (Figures 10G–I) showed partial metabolic normalization, with persistent alterations in ubiquinone/terpenoid-quinone biosynthesis and incomplete restoration of galactose metabolism. Collectively, these findings demonstrate that EEN therapy induces stage-specific metabolic reprogramming in CD patients, characterized by the activation of specialized metabolite production, partial correction of lipid metabolism abnormalities, and persistent modifications in neuroactive compound synthesis while maintaining differential regulation of energy-related pathways.

## Discussion

In this study, 16S rRNA gene sequencing was used to explore the relationship between the gut microbiota and EEN therapy. Our findings indicate that EEN significantly alters the gut microbiota structure in celiac patients, while overall alpha diversity remains relatively stable. This is consistent with previous studies, suggesting that EEN’s primary mechanism may involve reshaping microbial community structure rather than altering diversity (Diederen et al., 2020; Geesala et al., 2024). The consistent reduction in alpha diversity in both treatment and pre-treatment groups, when compared to healthy controls, aligns with previous research and reinforces the link between CD and a more imbalanced microbiome (Ambrozkiewicz et al., 2020; Pierce et al., 2024). Moreover, the significant differences in beta diversity across the groups demonstrate that EEN effectively modulates the composition of the gut microbiome.

In our analysis, we observed that *Fusobacterium, Veillonella, Acidaminococcus, Megasphaera,* and *Moryella* were enriched in the pre-treatment group. Previous studies have shown that *Fusobacterium nucleatum* is significantly enriched in the intestinal mucosa of CD patients, particularly in those with colonic CD, where its abundance correlates with disease activity and refractory behavior (Cao et al., 2020; Zhou et al., 2021). *Fusobacterium* exacerbates intestinal barrier disruption and inflammation by activating the endoplasmic reticulum stress (ERS) pathway and upregulating the expression of CARD3 (Cao et al., 2020). Alsulaiman et al. (2023) reported that the abundance of *Veillonella* is significantly elevated in CD patients and is positively correlated with intestinal inflammatory markers, such as calprotectin (Shi et al., 2022). Furthermore, the metabolites of *Veillonella* may exacerbate mucosal damage by reducing the anti-inflammatory effects of SCFAs (Arcila-Galvis et al., 2022). *Acidaminococcus* is involved in amino acid metabolism and glycolysis pathways and may contribute to the development of CD by altering the gut microenvironment (Oh et al., 2021). Xu et al. (2022) found that a high-fat diet can promote the proliferation of both *Fusobacterium* and *Acidaminococcus*. In contrast, the CD exclusion diet, when combined with partial enteral nutrition, has been shown to reduce the abundance of pro-inflammatory bacteria and restore microbial balance (Levine et al., 2019). Consistent with these findings, our study observed a reduction in the abundance of *Fusobacterium*, *Veillonella*, and *Acidaminococcus* following EEN therapy. This decrease may contribute to the therapeutic effects of EEN by alleviating intestinal inflammation and promoting mucosal healing, thus underscoring the potential role of these bacteria in the pathogenesis of CD.

Several key mechanisms underlying the therapeutic effects of EEN in CD have been identified in this study. ABC transporters play a crucial role in regulating metabolic processes, including lipid metabolism and energy balance, which, in turn, affect the function and repair capacity of intestinal cells (Thévenod and Lee, 2024). The activation of the pentose phosphate pathway not only provides precursors for nucleotide synthesis but also enhances cellular antioxidant capacity by generating NADPH (Jana et al., 2021). Additionally, L-seryl-tRNA(Sec) selenocysteine transferase, which is involved in selenoprotein synthesis, indirectly supports cellular antioxidant capacity and overall health in intestinal cells. The upregulation of this enzyme during EEN therapy likely contributes to the mitigation of oxidative stress in CD, creating a synergistic effect with other therapeutic mechanisms (Zheng et al., 2020). The gut microbiota also plays a crucial role in modulating gut barrier function through the metabolism of nucleotides and their derivatives. For instance, *Lactiplantibacillus plantarum* 12 has been shown to enhance gut barrier function in a murine model of colorectal cancer by regulating nicotinamide adenine dinucleotide metabolism (Zhao et al., 2023). Furthermore, NAD+ supplementation has been demonstrated to restore tight junction protein expression and improve gut barrier integrity in a high-fat diet-induced model of gut barrier damage, likely through the activation of the AMPKα/SIRT1 signaling pathway (Yamasaki et al., 2024). These findings suggest that EEN therapy may exert its therapeutic effects on CD via multiple mechanisms, including the modulation of host metabolism and gut microbiota composition.

Metabolomic profiling of plasma and fecal samples from CD patients before and after treatment reveals significant metabolic differences. Notable alterations were observed in several metabolic pathways following EEN therapy, including phenazine biosynthesis, indole diterpene alkaloid biosynthesis, sphingolipid signaling, and sphingolipid metabolism. Phenazine biosynthesis is one of the metabolic pathways affected by EEN therapy. Phenazines are a group of redox-active compounds that can induce the production of reactive oxygen species (ROS) (Xu et al., 2013). At low concentrations, phenazines may have beneficial effects by activating host antioxidant pathways, such as the nuclear factor erythroid 2-related factor 2 (Nrf2) pathway. Nrf2 is a transcription factor that regulates the expression of antioxidant and detoxification genes, helping to protect cells from oxidative damage (Saha et al., 2020). However, at higher concentrations, phenazines may exacerbate oxidative damage to the intestinal epithelium, potentially worsening CD symptoms (Peng et al., 2021). The modulation of phenazine biosynthesis by EEN therapy may therefore represent a delicate balance in regulating oxidative stress in CD patients. Indole diterpenes can indirectly support gut barrier function by inhibiting the growth of pathogenic bacteria, such as *Escherichia coli*, or by modulating the activity of host ion channels (Hou et al., 2022). The gut barrier function is critical in preventing the translocation of bacterial components and toxins into the intestinal mucosa, which can trigger immune responses and inflammation (Wang et al., 2023). By enhancing gut barrier function, indole diterpenes may help to reduce the permeability of the intestinal epithelium and prevent the infiltration of harmful substances, thereby alleviating CD symptoms (Ye et al., 2022). Sphingolipid metabolism and signaling are also significantly altered in CD patients. Sphingolipids are a class of bioactive lipids that play important roles in cell signaling, membrane structure, and inflammation regulation (Stith et al., 2019). Sphingosine-1-phosphate (S1P), a key sphingolipid metabolite, mediates lymphocyte egress from lymphoid tissues via the S1PR1 receptor. Elevated levels of S1P have been found in the intestines of CD patients and are associated with increased inflammatory cell infiltration and tissue damage (Zou et al., 2023). EEN therapy appears to modulate sphingolipid metabolism, potentially reducing S1P levels and thereby decreasing inflammatory infiltration and tissue injury in the gut. These findings suggest that EEN therapy may improve the metabolic status of CD patients by modulating key metabolic pathways.

Through the comprehensive integration of microbiome and metabolome analyses, our study provides valuable insights into how EEN therapy impacts CD, offering a foundation for future research. Subsequent studies could explore the specific mechanisms and interactions between distinct microbiota, genetic pathways, and metabolic pathways to deepen our understanding of the role of EEN therapy in treating CD. Additionally, investigating the long-term effects of EEN therapy on CD patients, especially when combined with other treatments such as pharmacotherapy and surgery, will be crucial for future research. By conducting comprehensive, multidisciplinary research, we can further enhance our understanding of the potential benefits of EEN therapy in CD treatment, ultimately providing more scientifically grounded and effective clinical guidance.

### Limitations and future directions

Although this study provides valuable insights into the effects of EEN on the gut microbiome and metabolome in CD, its cross-sectional design limits the ability to draw definitive conclusions regarding causality. Future research should focus on longitudinal, prospective studies with adequately powered sample sizes to confirm these findings and explore the long-term effects of EEN on microbial and metabolic profiles. Furthermore, mechanistic studies are essential to elucidate how specific microbial taxa and metabolites interact with host immune and metabolic pathways to facilitate disease remission. In addition, functional validation studies can confirm the role of these altered metabolites in the pathogenesis of CD and their association with inflammation. Finally, the development of personalized nutritional therapies tailored to individual microbial and metabolic signatures holds great promise for improving therapeutic outcomes in CD patients. Such approaches could ultimately lead to more precise and effective disease management strategies.

## Conclusion

Our research thoroughly integrates microbiome and metabolome analysis, laying a scientific foundation for optimizing nutritional strategies in CD management. EEN therapy, which effectively induces remission without the adverse effects commonly associated with corticosteroids, holds promise for further refinement through the targeted modulation of specific microbial and metabolic pathways. For example, dietary interventions or probiotics designed to reduce the abundance of pro-inflammatory bacteria or enhance the production of anti-inflammatory metabolites could be explored as adjunctive therapeutic options. Additionally, the study highlights the potential of microbiome and metabolome profiling as biomarkers for monitoring disease activity and assessing treatment response in CD patients.

In summary, this study enhances our understanding of the complex interactions between gut microbiota, host metabolism, and EEN therapy in CD. By elucidating the regulatory mechanisms underlying EEN-induced remission, our findings offer valuable insights for the development of more effective and targeted therapeutic strategies for CD patients. The identification of specific microbial and metabolic pathways provides a robust framework for future research and clinical applications aimed at improving disease management and patient outcomes.

## Data Availability

The high-throughput sequence data have been deposited in the National Center for Biotechnology Information (NCBI) BioProject database with project number PRJNA1285314. All other data are available upon request from the authors.

## Footnotes

^1^ https://docs.qiime2.org/2022.11/tutorials/feature-classifier/

^2^ https://metacyc.org/
